# Video-EEG/polygraphy in status epilepticus

**DOI:** 10.3389/fneur.2026.1776158

**Published:** 2026-02-18

**Authors:** Giuseppe d’Orsi, Maria Teresa Di Claudio, Umberto Costantino, Carmela Pia Ferro

**Affiliations:** Neurology Unit – Epilepsy Center, Fondazione IRCCS Casa Sollievo della Sofferenza, San Giovanni Rotondo (FG), Italy

**Keywords:** differential diagnosis, nonconvulsive status epilepticus, refractory status epilepticus, status epilepticus, video-EEG/polygraphy

## Abstract

**Background:**

The management of Status Epilepticus (SE) is anchored by time-dependent protocols, recognizing that time is the primary prognostic determinant. Acute care application is complicated by critical diagnostic ambiguities, risking delays and therapeutic errors. This narrative analysis highlights the role of continuous Video-EEG/Polygraphy (V-EEG/PG) in resolving key practical challenges in SE management.

**Methods:**

We conducted a retrospective narrative analysis of five illustrative clinical vignettes from a tertiary-care center. Cases were selected to address the three major management challenges: Time (Nonconvulsive SE vs. stupor), Etiology (SE vs. Toxic-Metabolic Encephalopathies), and Differential Diagnosis (seizures vs. behavioral mimics). The V-EEG/PG utility was assessed based on its capacity to provide objective electro-clinical correlation, guiding timely therapeutic escalation or de-escalation.

**Results:**

V-EEG/PG provided critical clinical insights by: (1) Objectively Confirming Cessation, preventing silent progression to Refractory SE. (2) Guiding Etiological Triage, aiding in the exclusion of SE/NCSE in metabolic disease, avoiding the harmful use of high-dose Anti-Seizure Medications and unnecessary anesthetic exposure. (3) Ensuring Diagnostic Accuracy, serving as highly reliable standard for differentiating true SE from mimics, thus safeguarding patients from chronic misdiagnosis and pharmacological toxicity.

**Conclusion:**

Effective SE management should ideally incorporate a shift from a purely chronological approach to an electro-clinical strategy. Continuous V-EEG/PG is a pivotal diagnostic asset that resolves key ambiguities. The evidence suggests that V-EEG/PG should be considered a standard of care in specific high-risk and ambiguous acute care settings, particularly in patients with stupor/coma post-treatment, altered mental status in metabolic disease, or prolonged, non-stereotyped motor events.

## Introduction

**Figure 1:**
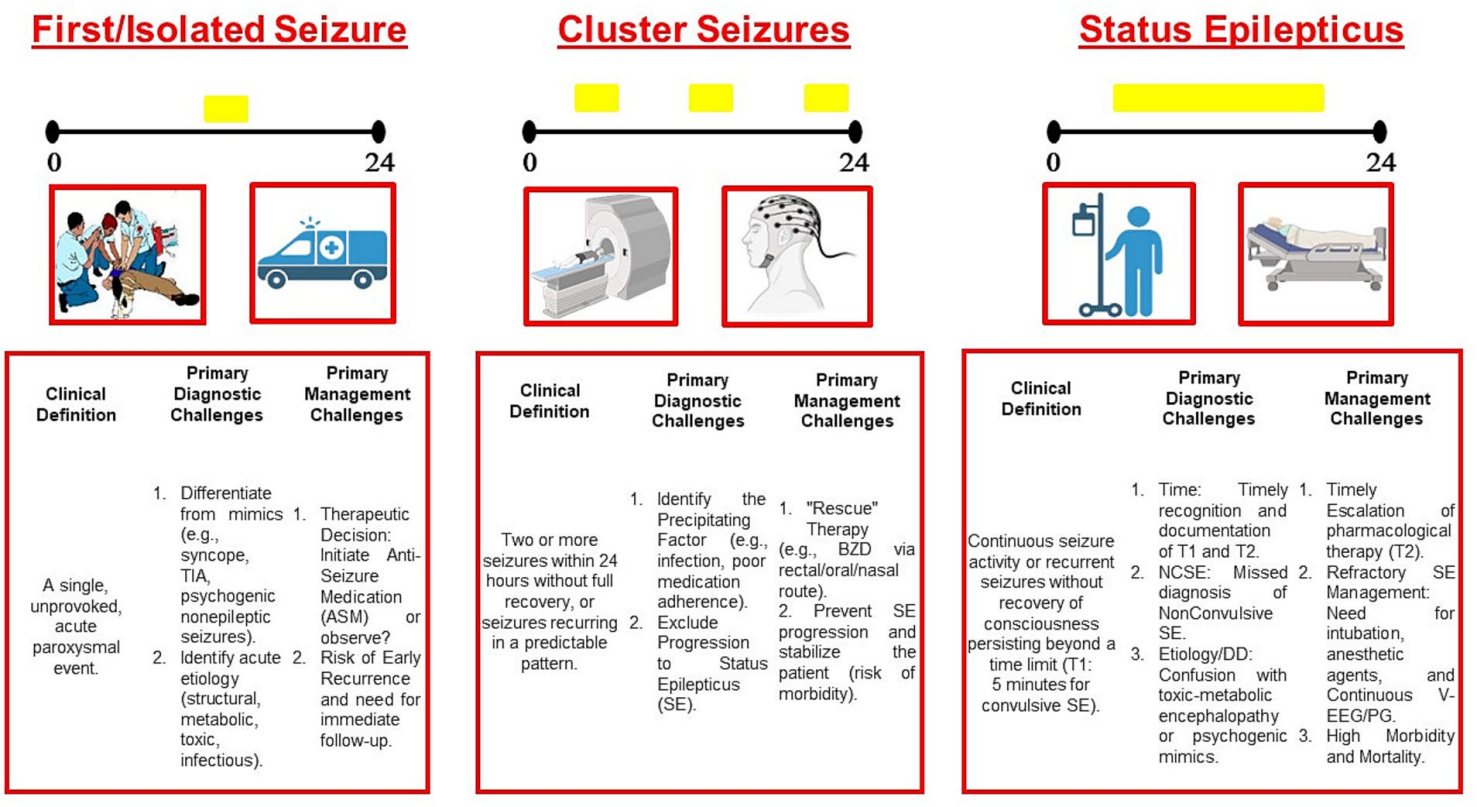
Challenges in epileptological emergencies: three main epileptological emergencies within a 24-h timeline, highlighting the clinical definition, primary diagnostic challenges, and primary management challenges for each condition.

Epileptic seizures represent a frequent cause for Emergency Department (ED) admission, manifesting as isolated paroxysmal events, cluster seizures, or prolonged, self-sustaining activity known as Status Epilepticus (SE). While isolated seizures primarily constitute a diagnostic emergency, cluster seizures and SE demand immediate attention as both diagnostic and therapeutic emergencies. Achieving the goal of timely, appropriate, and effective pharmacological treatment, guided by prompt diagnosis, necessitates a robust structural and multidisciplinary approach. This collaboration must span from the pre-hospital setting (Emergency Medical Services) to the hospital (ED, neurology and specialized wards, and Intensive Care Unit), involving key players such as emergency physicians, neurologists, and intensivists. Furthermore, there is a critical need for rapid-acting intravenous (IV) anti-seizure medications (ASMs) to quickly achieve seizure control, preventing the progression of a diagnostic urgency into a severe therapeutic crisis. Time is, therefore, the primary and most critical factor. In fact, the International League Against Epilepsy (ILAE) emphasizes that the principle of “Time is Brain,” traditionally reserved by neurologists for the management of acute ischemic stroke, applies equally to SE (1). Because prognosis significantly worsens with increasing duration of critical activity (2–4), all major international therapeutic protocols, guidelines, and recommendations (5–7) recognize a staged approach using different pharmacological lines based on specific time points, which further emphasizes the necessity of prompt recognition and treatment to achieve rapid seizure control and mitigate long-term consequences: Early SE (first line: Benzodiazepines), Established SE (second line: Anti-Seizure Medication, ASM), and Refractory SE (third line: Anesthetics or other ASMs). Refractory Status Epilepticus (RSE) is classically defined as seizure activity that persists beyond 30 min despite the administration of first- and second-line treatments. This established theoretical framework, however, often encounters critical divergence from the complexities encountered in real-world clinical practice. Notably, the transition from second-line ASMs to third-line anesthetic therapy is not always a straightforward chronological escalation. Especially in cases of Non-Convulsive Status Epilepticus (NCSE), or in complex patients with significant medical comorbidities (e.g., extensive ischemic stroke or metabolic failure), the decision to initiate general anesthesia must be carefully weighed against the patient’s frailty and the risk–benefit ratio of aggressive treatment. In such scenarios, a strictly time-dependent paradigm may be secondary to a more individualized approach, particularly where access to Intensive Care Unit (ICU) resources is limited. Beyond the primary urgency of time-to-treatment, the management of SE is critically challenged by three core issues: the difficulty of assessing the true duration of the epileptic activity, the identification of the underlying etiology, and the need for accurate differential diagnosis (Figure 1). Through a series of presented clinical vignettes, we aim to illustrate how these three principal challenges complicate the transition from theoretical guidelines to effective bedside management, and also the role of continuous Video-EEG/Polypgraphy (V-EEG/PG) monitoring in the acute emergency setting to resolve these ambiguities and drive timely, appropriate therapeutic decisions.

## Methods

This study employs a narrative analysis approach based on a retrospective series of clinical cases. The primary goal is to provide a practical, evidence-based discussion of the diagnostic and management challenges encountered in SE in a tertiary-care setting, specifically highlighting the role of Video-EEG/PG in resolving these challenges. We retrospectively reviewed the clinical records of all adult patients admitted to the Neurology and ICU at Fondazione IRCCS Casa Sollievo della Sofferenza between May 2022 and December 2025 who met the criteria for:

- SE, as defined by the ILAE (6).
- RSE, defined as SE persisting despite the administration of adequate doses of a benzodiazepine and a second-line ASM.
- NCSE is defined according to the Salzburg Criteria, requiring a combination of specific electrographic patterns and clinical response to ASMs or typical evolution. Patterns that do not strictly fulfill ictal criteria but are not clearly interictal are classified within the Ictal-Interictal Continuum (IIC), as per the 2021 ACNS nomenclature.

Cases were purposefully selected to represent the most common and instructive diagnostic and therapeutic challenges where the clinical picture was ambiguous or refractory, necessitating Video-EEG/PG for definitive diagnosis, differential diagnosis, and therapeutic guidance. Data collected for each selected case included: demographics, etiology of SE, detailed pharmacological treatment timelines, V-EEG/PG findings (including ictal patterns and duration), clinical course, and final outcome (e.g., duration of ICU stay, neurological status at discharge). Continuous V-EEG/PG monitoring was performed using the EEG System Plus Micromed with a minimum of 21 scalp electrodes placed according to the International 10–20 system. Polygraphy leads were systematically applied to specific muscle groups (e.g., masseter, deltoid, tibialis anterior) and vital parameters (ECG, respiration) to assist in the differential diagnosis between epileptic events and non-epileptic events (e.g., functional or movement disorders). V-EEG/PG analysis was performed by board-certified epileptologists, focusing on identification of ictal/inter-ictal patterns, correlation between electrical activity and clinical semiology, and assessment of background activity suppression in sedated patients. The selected cases were analyzed narratively to extract key clinical lessons and decision-making algorithms. The analysis was specifically focused on the three V-EEG/PG use in acute setting: 1- the Challenge of Time: assessing the utility of V-EEG/PG in differentiating between post-treatment stupor and ongoing NCSE, and confirming the objective seizure termination time; 2- the Challenge of Etiology: analyzing the role of V-EEG/PG in confidently excluding SE/NCSE in Toxic-Metabolic Encephalopathies, thereby guiding etiological treatment and preventing toxic overtreatment; 3- the Challenge of Differential Diagnosis: determining the value of V-EEG/PG as the objective gold standard for electro-clinical correlation in distinguishing true seizures from behavioral mimics.

## Results

### The challenge of time

#### Case 1

A 72-year-old female patient with a history of hypertension and atrial fibrillation was admitted to our Neurology Unit 12 h after sustaining an acute ischemic stroke in the left middle cerebral artery territory. Over the course of 24 h, she experienced three right focal motor seizures. The third seizure, lasting 4 min, was aborted with an IV dose of diazepam 10 mg (first line). A V-EEG-PG, initiated before and during drug administration, documented a gradual disappearance of both the clinical and subsequent electrographic component (Supplementary Figure S1). Following treatment, the patient appeared deeply soporific (GCS 8), without overt convulsive movements. The emergency neurologist initially attributed the patient’s sopor to a combination of post-ictal phase and diazepam sedation. Given the apparent clinical control of the motor seizures, the decision was made to wait, assuming seizure termination, and immediately discontinue the V-EEG/PG recording. The patient’s neurological status was instead monitored clinically for 8 h. When the patient’s status remained unchanged after 8 h, a standard 30 min EEG was finally ordered. The EEG immediately disclosed the presence of continuous, rhythmic, and recruiting epileptiform abnormalities, predominant over the left frontotemporal leads, diagnostic of NCSE. The administration of a second IV dose of diazepam 10 mg, followed by a loading dose of levetiracetam 2,000 mg (second line), failed to produce electro-clinical improvement. Although 2,000 mg is within the therapeutic range, in the context of prolonged NCSE, this dose might have been insufficient to achieve rapid control. The true duration of the continuous epileptic activity, spanning over 8 h, coupled with the lack of response to delayed second-line therapy, pushed the patient well into RSE. This significant delay, compounded by the gradual onset of respiratory compromise, necessitated immediate intubation, transfer to the Intensive Care Unit (ICU), and the initiation of high-dose anesthetic therapy.

#### Case 2

A 68-year-old male patient presented with a clinical picture initially indistinguishable from that of Case 1, having experienced two right focal motor seizures following an acute ischemic stroke in the left middle cerebral artery territory. The third seizure, lasting 3 min, was aborted with an IV dose of diazepam 10 mg. Post-treatment, the patient remained soporific (GCS 8). Given the ambiguity of the sopor and the high risk of NCSE in the acute neurological setting, continuous V-EEG/PG monitoring was initiated immediately before and —unlike the previous case—maintained continuously following the diazepam administration. This strategic choice quickly disclosed that the persistent sopor was not attributable to drug effects. While the bedside clinical examination suggested only a non-specific post-ictal state (as in Case 1), the synchronized video recording provided a critical diagnostic advantage, revealing a mild rightward eye deviation that was evident only upon retrospective review of the footage. This subtle clinical sign was perfectly synchronized with focal, continuous rapid and rhythmic activity, predominantly over the left frontotemporal leads. This electrographic pattern allowed for the definitive diagnosis of Established SE within the first hour of observation, preventing the diagnostic “blind spot” encountered in Case 1. With the objective V-EEG/PG confirmation of ongoing SE, the emergency neurologist immediately administered the second-line ASM, lacosamide 400 mg. Seizure control was achieved rapidly and confirmed by the immediate electrographic clearance of the ictal pattern on the V-EEG/PG. By avoiding the 8-h delay seen in Case 1, the patient’s total duration of SE was significantly reduced, resulting in a better neurological outcome and the successful avoidance of escalation to anesthetic agents and ICU admission (Supplementary Figure S2).

### The challenge of etiology

#### Case 3

**Figure 2:**
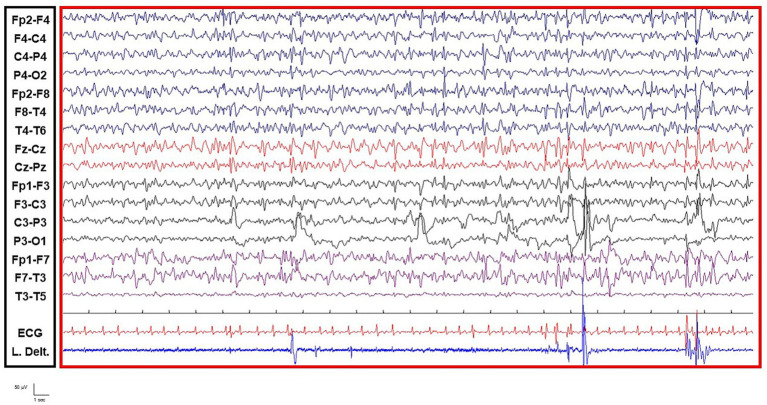
V-EEG/polygraphy differentiating metabolic myoclonus from status epilepticus (Case 3). The EEG shows diffuse background slowing and intermittent, diffuse sharp waves. The EMG recording (left deltoid muscle) demonstrates myoclonic jerks that are not time-locked to the transient epileptiform activity, definitively ruling out a cortical seizure origin (status epilepticus). This diagnostic clarification allowed for the discontinuation of anti-seizure medications and the initiation of emergency dialysis, leading to full clinical recovery.

A 75-year-old female with severe chronic renal failure was admitted to our ED with marked confusion (GCS 10) and intermittent, generalized myoclonic jerks. Initial lab tests indicated severe uremia. Despite the primary metabolic picture, due to the high clinical suspicion of convulsive SE (CSE), the patient was empirically loaded with Levetiracetam 1,500 mg before V-EEG/PG was available. Following this intervention, the patient’s confusion worsened, and the myoclonic jerks persisted. When the pharmacological intervention failed and the patient’s status deteriorated, V-EEG/PG monitoring was initiated. The EEG showed a diffuse background slowing, with intermittent, diffuse sharp waves. Importantly, no continuous ictal patterns were identified. While the absence of rhythmic or periodic patterns already made a diagnosis of NCSE unlikely based on the baseline EEG alone, the PG demonstrated that the myoclonic jerks were not time-locked to the transient epileptiform activity, definitively confirming they were primarily of subcortical/metabolic origin. Based on the V-EEG/PG findings, the emergency neurologist definitively ruled out SE/NCSE. The focus immediately shifted to the etiological treatment. The Levetiracetam was discontinued, and the patient was started on emergency dialysis and aggressive metabolic correction. The patient’s consciousness and myoclonic jerks rapidly resolved only after the metabolic derangement was corrected (Figure 2; Supplementary Video 1).

#### Case 4

**Figure 3:**
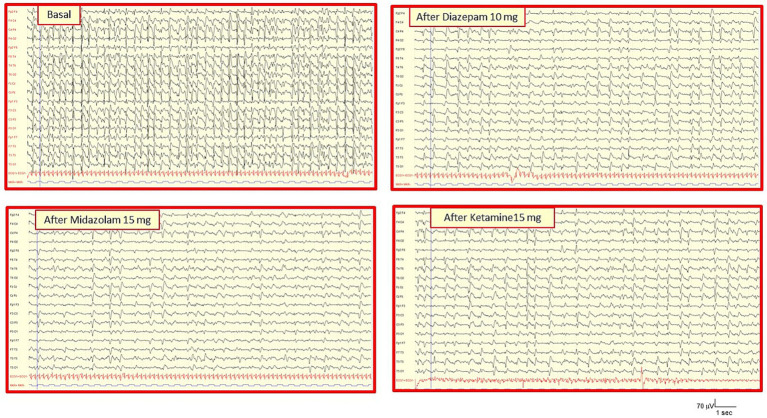
V-EEG/PG distinguish metabolic mimicry from NCSE (Case 4). The basal EEG (upper tracing) shows severe diffuse background slowing with intermittent triphasic waves and periodic discharges. In the absence of clinical motor signs (metabolic coma), this pattern was diagnosed as NCSE. Nevertheless, continuous V-EEG/PG monitoring demonstrated that these periodic patterns did not evolve, did not propagate, and were non-responsive to multiple anti-seizure medication trials (e.g., diazepam 10 mg, midazolam 15 mg, ketamine 2 mg/Kg bolus). Finally, V-EEG/PG provided the objective proof required to confidently exclude NCSE. This allowed the management team to prioritize aggressive etiological correction over potentially harmful pharmacological treatment, acting as a critical triage tool to prevent the overtreatment of a systemic disease.

A 37-year-old patient with Type 1 Diabetes Mellitus was admitted in a comatose state (GCS 7) due to severe Diabetic Ketoacidosis (DKA). Initial standard EEG showed severe diffuse background slowing with Generalized Periodic Discharges (GPDs) characterized by a triphasic morphology at a frequency of 1–1.25 Hz. According to the Salzburg criteria, this pattern did not fulfill the requirements for definite NCSE but rather fell within the Ictal-Interictal Continuum (IIC). Continuous V-EEG/PG monitoring showed that these periodic patterns did not evolve in frequency or morphology and did not propagate. Despite various therapeutic trials (e.g., diazepam 10 mg, midazolam 15 mg, lacosamide 200 mg), the EEG remained largely unchanged. Although these ASM doses may be considered relatively low for a definitive “treatment trial” in SE, the lack of any transient modification in the GPDs, combined with the severe metabolic derangement, strongly argued against a true RSE/NCSE (Figure 3). The decision to rule out NCSE relied on the integrated assessment of the clinical context, the lack of electro-clinical evolution on continuous monitoring, and the subsequent response to etiological treatment. The ICU team maintained focus on aggressive fluid treatment and insulin therapy for the DKA, confidently avoiding further escalation to high-dose anesthetics. As the metabolic parameters (pH, ketones) improved, the periodic EEG discharges gradually disappeared, and the patient emerged from the coma.

### The challenge of differential diagnosis

#### Case 5

A 65-year-old female patient presented to another Emergency Department 10 years prior with recurrent motor episodes of a bizarre semiology (e.g., asynchronous clonic movements and fluctuating responsiveness). These episodes often lasted up to 30 min, and though they appeared to temporarily subside after a diazepam 10 mg bolus, they would typically recur within 15 min and persist for several hours. During the initial assessment, the patient underwent a standard, short-duration EEG. A spontaneous motor episode occurred during the recording. Lacking simultaneous video documentation, movement artifacts were erroneously considered paroxysmal epileptiform abnormalities, and the episode was misinterpreted as an epileptic seizure. A diagnosis of Epilepsy was made, and the patient was immediately started on Valproic Acid 1,000 mg/day. Despite the initial therapy, the patient continued to experience prolonged motor episodes of analogous semiology and repeatedly presented to various EDs. The successive emergency neurologists failed to revise the initial diagnosis, leading to increased dosages and the subsequent addition of multiple ASMs (including Levetiracetam 2000 mg, Lacosamide 400 mg) over the following decade. This resulted in a significant burden of polypharmacy and side effects (e.g., sedation, weight gain). Following 10 years of intractable “seizures,” the patient was finally admitted for prolonged V-EEG/PG monitoring as part of a reassessment for drug-refractory epilepsy. During multiple recorded, typical, and prolonged episodes, the video confirmed a semiology characteristic of functional episodes (PNES) (e.g., non-stereotyped movements, sustained eye closure). Crucially, the simultaneous EEG trace showed no concurrent epileptiform discharges and a preserved background rhythm. All ASMs were gradually and safely withdrawn. The patient was referred for appropriate psychiatric and psychological treatment. The cessation of polypharmacy significantly improved her quality of life (Supplementary Figure S3; Supplementary Video 2).

## Discussion

The management of SE is anchored by the protocol principle that “Time is Brain” (1). However, the five clinical vignettes collectively reveal a critical vulnerability in this approach: blind adherence to time-based guidelines, decoupled from objective data, risks diagnostic delay or therapeutic error. The central lesson is the indivisible duality required between clinical assessment and continuous electrophysiological monitoring. While clinical signs (stupor, myoclonus) must initiate care, they cannot independently validate therapeutic success. The utility of V-EEG/PG is not merely diagnostic but triage-defining, allowing the clinician to manage the patient’s *actual electrophysiological state*. The vignettes highlight V-EEG/PG’s critical role in resolving the three major obstacles to timely and appropriate therapy.

### The challenge of time

The failure to recognize NCSE remains the most significant temporal challenge. Clinical stupor, especially post-benzodiazepine or post-ictal, often causes electro-clinical decoupling, falsely signaling seizure termination. In fact, Case 1 highlights a significant clinical pitfall: the risk of assuming seizure termination based solely on the cessation of motor activity. The 8-h delay in identifying NCSE and the subsequent need for aggressive ICU management underscore why V-EEG/PG should not be discontinued prematurely in high-risk patients with structural brain injury, where the clinical exam (GCS 8) is confounded by multiple factors. Our analysis strongly supports that any persistent alteration of consciousness in high-risk patients must be considered NCSE until proven otherwise. Continuous V-EEG/PG is not merely a diagnostic adjunct; its use is strongly recommended for diagnosing and monitoring treatment effectiveness and confirming the true time of seizure termination, preventing the silent progression to RSE that occurred in Case 2. The essential integration of clinical status with continuous EEG data allows for the accurate characterization of the ongoing critical event, thereby guiding definitive and time-sensitive therapy. Finally, our findings on the risks of untreated NCSE align with several consensus statements that advocate for continuous EEG monitoring in all comatose patients and those with altered mental status after seizure termination (8, 9). This emphasis is particularly crucial in the ICU setting, where subtle signs are often masked by sedation, a topic widely discussed in the literature (10).

### The challenge of etiology

Case 3 and 4 highlight the critical need for V-EEG/PG in the setting of non-motor manifestations, such as Toxic-Metabolic Encephalopathies. While the presence of continuous ictal activity mandates immediate SE treatment, V-EEG/PG is pivotal for confidently excluding true SE/NCSE when movements (myoclonus) or EEG anomalies (periodic patterns) are ambiguous (11). The V-EEG/PG serves three key functions:

1. Differential Diagnosis: Polygraphy differentiates non-epileptic, subcortical metabolic myoclonus from true cortical seizures, preventing the unnecessary escalation of ASMs (Case 3). Specifically, in Case 3, PG provided definitive objective confirmation that the jerks were of subcortical/metabolic origin. It is important to acknowledge that in real-world clinical settings, metabolic myoclonus can occasionally present with rhythmic or stereotyped features that may mimic focal motor seizures. In this instance, V-EEG/PG served as the “gold standard” to avoid the common pitfall of over-treating a metabolic encephalopathy with potentially neurotoxic ASM doses.
2. Therapeutic Triage: Continuous monitoring demonstrates that ambiguous or periodic EEG patterns in metabolic contexts are often non-evolving and non-responsive to ASMs, justifying a conservative approach toward antiseizure therapy (Case 4). However, it is crucial to recognize that true NCSE can occasionally be triggered by metabolic or toxic derangements. Even in these instances, the cornerstone of management remains the aggressive treatment of the underlying cause (e.g., organ failure, ketoacidosis), which often proves more effective than aggressive ASM escalation or anesthetic intervention.
3. Prevention of Overtreatment: By integrating clinical status with continuous EEG data, V-EEG/PG allows clinicians to prioritize etiological correction while avoiding the risks associated with unnecessary therapeutic over-escalation, invasive procedures, and pharmacological toxicity.

### The challenge of differential diagnosis

Case 5 illustrates that the failure to perform adequate electro-clinical correlation using V-EEG/PG at the time of the initial diagnosis leads to a potentially multi-decade cascading diagnostic error. Distinguishing true epileptic seizures from their behavioral mimics is impossible based on clinical description or standard EEG alone (12), particularly if the clinical picture and the EEG are not correlated, and especially if the EEG is used to guide the clinical assessment and not vice-versa. V-EEG/PG is the objective gold standard that can prevent the misdiagnosis of functional episodes as Refractory Epilepsy and RSE, thereby serves as the gatekeeper against lifelong diagnostic error safeguarding the patient from unnecessary pharmacological toxicity and ensuring timely access to effective psychological intervention. Nevertheless, it should be noted that in many such cases, a detailed reassessment of the clinical history— focusing on the absence of stereotypy and the presence of specific triggers—along with the review of smartphone-recorded videos by family members, may raise a strong suspicion of PNES even before formal testing. In this context, V-EEG/PG served not as the exclusive diagnostic clue, but as the definitive confirmatory evidence needed to resolve a decade of diagnostic uncertainty.

## Indications of continuous V-EEG/PG in urgency/emergency epilepsy

The clinical evidence derived from these five vignettes strongly advocates for the reclassification of continuous V-EEG/PG from an optional ancillary tool to a pivotal diagnostic tool in specific high-risk acute care settings (Tables 1, 2). This shift ensures a transition from a purely chronological or clinical assessment to a V-EEG/PG-guided electro-clinical strategy. We propose the routine implementation of continuous V-EEG/PG monitoring for all patients presenting with the following three key indications:

1. Stupor or coma following initial seizure termination (suspicion of NCSE). As demonstrated in the comparison between Cases 1 and 2, clinical judgment is often an insufficient endpoint for confirming the cessation of SE. V-EEG/PG is the only objective tool capable of distinguishing post-ictal stupor or drug-induced sedation from ongoing NCSE. Early detection through monitoring prevents the silent progression to RSE.
2. Altered mental status in the context of systemic or metabolic disease. In patients with acute neurological insults (e.g., ischemic stroke) or systemic failures (e.g., uremia, ketoacidosis), mental status changes are frequently multifactorial. V-EEG/PG serves as a critical triage tool to determine if a comatose state is purely metabolic or co-exists with occult NCSE. This prevents the misclassification and overtreatment of systemic encephalopathy-induced EEG anomalies (e.g., triphasic waves).
3. Prolonged, non-stereotyped motor events (suspicion of PNES or metabolic myoclonus). When paroxysmal motor episodes resist first-line benzodiazepines, the diagnostic priority shifts to etiology. The addition of PG is essential to differentiate true cortical seizures from subcortical metabolic myoclonus or PNES. As highlighted in Case 5, the absence of synchronized video-EEG can lead to a decade of diagnostic error and toxic polypharmacy due to the misinterpretation of muscle artifacts as ictal activity.

**Table 1:** V-EEG/PG indications, challenges, and clinical rationales.

Indication for V-EEG/PG	Underlying challenge	Rationale for V-EEG/PG Use
Persistent altered mental status post-seizure	Challenge of time: Distinguishing post-ictal stupor/sedation from ongoing nonconvulsive status epilepticus (NCSE).	V-EEG is the *only* objective tool to confirm true seizure cessation, thereby preventing the silent progression to Refractory SE (RSE).
Ambiguous repetitive movements	Challenge of etiology and differential diagnosis: Distinguishing true epileptic activity from metabolic myoclonus, tremors, or psychogenic non-epileptic seizures (PNES).	Polygraphy (PG) differentiates cortical seizures from subcortical/non-epileptic movements. V-EEG ensures patients avoid unnecessary, potentially toxic Anti-Seizure Medications (ASMs).
Non-responsive SE (suspected RSE)	Challenge of time/therapy: Determining failure of first- and second-line treatments when clinical response is subtle or masked.	Mandatory for monitoring treatment efficacy in real-time. Confirms electro-clinical decoupling and guides urgent transition to third-line anesthetic therapy.
Encephalopathy of unknown etiology (coma)	Challenge of etiology: Determining if the comatose state is solely due to systemic/metabolic dysfunction or co-exists with occult NCSE.	Prevents the misclassification and overtreatment of purely metabolic-induced EEG anomalies by confirming the absence of sustained ictal activity.
Guiding anesthetic weaning (ICU)	Challenge of therapy: Titration and withdrawal of continuous intravenous anesthetics (e.g., propofol, midazolam).	Essential for titrating doses to a target pattern (e.g., burst suppression)

**Table 2:** Synoptic overview of clinical vignettes and the role of V-EEG/PG.

Case	Clinical challenge	V-EEG/PG finding	Final diagnosis	Key take-home message
1	Lack of recovery after SE	Subtle GPDs missed by standard EEG	NCSE	Avoid “diagnostic gap” by maintaining monitoring until recovery.
2	Subtle ocular movements	Synchronized V-EEG detected ictal activity	Established SE	V-EEG/PG allows for immediate triage and aggressive treatment.
3	Rhythmic jerks in uremia	No time-locking between EEG and myoclonus	Metabolic Myoclonus	Polygraphy prevents overtreatment with neurotoxic ASMs.
4	Coma with periodic EEG	Non-evolving GPDs (IIC pattern)	Metabolic Encephalopathy	Prioritize etiological treatment over ASM escalation in GPDs.
5	Refractory motor episodes	Normal EEG during typical events	PNES	V-EEG/PG is the gold standard to resolve long-term misdiagnosis.

Structured summary of the clinical challenges presented in each case, the specific neurophysiological findings obtained through continuous monitoring, and the consequent impact on diagnostic accuracy and therapeutic decision-making. ASM, antiseizure medication; GPDs, generalized periodic discharges; IIC, ictal-interictal continuum; NCSE, non-convulsive status epilepticus; PNES, psychogenic non-epileptic seizures; SE, status epilepticus; V-EEG/PG, video-electroencephalography/polygraphy.

The adoption of a V-EEG/PG-guided strategy provides a dual safeguard in the Emergency Department and ICU. It protects patients from the irreversible neurological damage caused by untreated, occult status epilepticus while simultaneously shielding them from the significant morbidity associated with unnecessary, high-dose anti-seizure medications and anesthetic toxicity. Therefore, continuous monitoring is no longer a luxury of specialized centers but a fundamental requirement for the safe and effective management of the critically ill neurological patient.

## Limitations

This study presents a narrative analysis based on a retrospective series of five selected case vignettes. While highly effective for demonstrating key clinical dilemmas and highlighting the indispensable role of V-EEG/PG, this methodological approach inherently carries several limitations that should be acknowledged. The cases were purposefully selected because they were instructive and unambiguous in illustrating the diagnostic and therapeutic utility of V-EEG/PG. This limits the generalizability of the findings compared to a large, prospective, consecutive cohort study. As a narrative case series, the study is descriptive and lacks the statistical power to formally quantify the efficacy of V-EEG/PG in reducing morbidity, mortality, or length of stay compared to standard care protocols. Moreover, the data reflects the specific protocols and resources of a single tertiary-care center. Resource limitations in other settings (e.g., availability of 24/7 V-EEG/PG monitoring or specialized epileptologists) may influence the direct applicability of our proposed. Finally, the analysis relies on data collected retrospectively, which may be subject to incomplete documentation regarding subtle clinical signs or exact pharmacological administration times, although V-EEG/PG data is inherently objective. Despite these limitations, this narrative analysis provides strong, practical evidence supporting the role of continuous V-EEG/PG as a pivotal triage tool for resolving the core ambiguities of Time, Etiology, and Differential Diagnosis in acute SE management.

## Conclusion

The clinical vignettes presented in this narrative analysis confirm that the effective management of SE frequently diverges from strict, time-based theoretical protocols due to critical ambiguities encountered in acute care. The three major obstacles to timely and appropriate therapy are the misidentification of the true duration of seizure activity (Time/NCSE), the confusion between epileptic and non-epileptic causes of altered mental status (Etiology), and the misdiagnosis of clinical mimics (Differential Diagnosis). The core finding is that continuous Video-EEG/PG is not merely an adjunct but the indispensable tool that resolves these diagnostic ambiguities. V-EEG/PG allows clinicians to:

1. Objectively Confirm Cessation: Distinguish post-ictal or drug-induced sopor from silent NCSE, preventing the critical delay that leads to RSE.
2. Guide Etiological Triage: Confidently exclude SE/NCSE in cases of Toxic-Metabolic Encephalopathy, preventing the unnecessary and harmful use of high-dose ASMs and prioritizing life-saving systemic correction.
3. Ensure Diagnostic Accuracy: Serve as the gold standard for differentiating true SE from its mimics, such as functional episodes and metabolic movements, thereby protecting patients from years of pharmacological toxicity and resource wastage. Specifically, the PG component provides the necessary electro-clinical correlation to distinguish cortical from subcortical phenomena.

In this light, while we acknowledge the organizational and logistical challenges of implementing V-EEG/PG in the ED or non-specialized ICUs, we argue that achieving this capability must be a primary objective in the era of precision medicine. The current “one-size-fits-all” approach to suspected SE often leads to diagnostic delays or dangerous overtreatment. From a health-economic perspective, the initial costs associated with equipment and specialized staffing are largely offset by the prevention of unnecessary and expensive procedures. As demonstrated in our cases, an accurate and early diagnosis prevents unjustified admissions to the ICU, the use of costly anesthetic agents, and the complications associated with prolonged hospitalization and pharmacological toxicity. The limited use of V-EEG/PG today is often not due to the cost of the technology itself, which is relatively contained, but rather to a lack of specific clinical training. The methodology remains underutilized because it is still poorly understood by much of the frontline emergency staff. Therefore, investing in the specialization of neurologists and technicians and fostering a culture of electro-clinical correlation through V-EEG/PG is a necessary step. The systematic integration of polygraphy into monitoring protocols allows for a transition from a “trial-and-error” model to an evidence-based, precision-driven diagnostic framework.

## Data Availability

The raw data supporting the conclusions of this article will be made available by the authors, without undue reservation.

## Supplementary material

SUPPLEMENTARY FIGURE S1 V-EEG/PG and the pitfall of premature discontinuation (Case 1). The V-EEG/PG trace illustrates continuous paroxysmal activity prevalent over the vertex and left superior-sylvian longitudinal derivations, with a corresponding myoclonic-type muscular potential recorded over the right deltoid muscle. Following the intravenous administration of diazepam 10 mg (indicated by the arrow), this electro-clinical pattern gradually disappeared, confirming seizure termination. However, the immediate discontinuation of monitoring prevented the detection of the subsequent re-emergence of NCSE. This highlights that V-EEG/PG must be continued in high-risk patients until the patient is fully awake.

SUPPLEMENTARY FIGURE S2 Successful detection of NCSE via continous V-EEG/polygraphy (Case 2). The V-EEG/PG documents paroxysmal activity prevalent over the left superior-sylvian derivations (Red Box), corresponding to the right focal motor seizure. Following intravenous diazepam 10 mg, the motor seizure ceases, but the V-EEG/PG reveals persistent, continuous focal rapid and rhythmic activity in the left soprasylvian leads (Blue Box). This activity was associated with mild rightward eye deviation (seen only on synchronized video), confirming the diagnosis of Established NCSE.

SUPPLEMENTARY FIGURE S3 Comparison between standard EEG (2015, left) and V-EEG/PG (2025, right) in a patient with psychogenic non-epileptic seizures (PNES). Both tracings show identical muscle artifacts during motor episodes. In 2015, the lack of simultaneous video documentation led to the erroneous interpretation of these artifacts as paroxysmal epileptiform activity, resulting in 10 years of unnecessary polypharmacy and drug-refractory “epilepsy.” In 2025, the V-EEG/PG recording definitively identified the same patterns as artifacts associated with functional movements, with a preserved background rhythm. This comparison highlights the “diagnostic trap” of movement artifacts in standard EEG. The use of V-EEG/PG was essential to correct the 10-year-old error, allow for the safe withdrawal of all anti-seizure medications, and significantly improve the patient’s quality of life.

SUPPLEMENTARY VIDEO 1 Case 3. The video shows a patient with severe chronic renal failure presenting with altered consciousness and arrhythmic jerks in the left upper limb. These clinical motor events were proven by simultaneous polygraphy (EMG) to be not time-locked to cortical EEG discharges. This confirms a subcortical metabolic origin, effectively ruling out convulsive status epilepticus.

SUPPLEMENTARY VIDEO 2 Case 5. The video captures rapid, arrhythmic, and non-stereotyped movements involving the chest and abdomen. Simultaneous EEG shows prominent muscle and movement artifacts that were historically misinterpreted as epileptiform activity. This case highlights the critical role of V-EEG in ensuring diagnostic accuracy and preventing long-term pharmacological toxicity.
