# Supplementary figures and images for: Video-EEG/polygraphy in status epilepticus
Source: Front Neurol. 2026 Feb 18;17:1776158. doi: 10.3389/fneur.2026.1776158 (PMC12956651; doi:10.3389/fneur.2026.1776158)

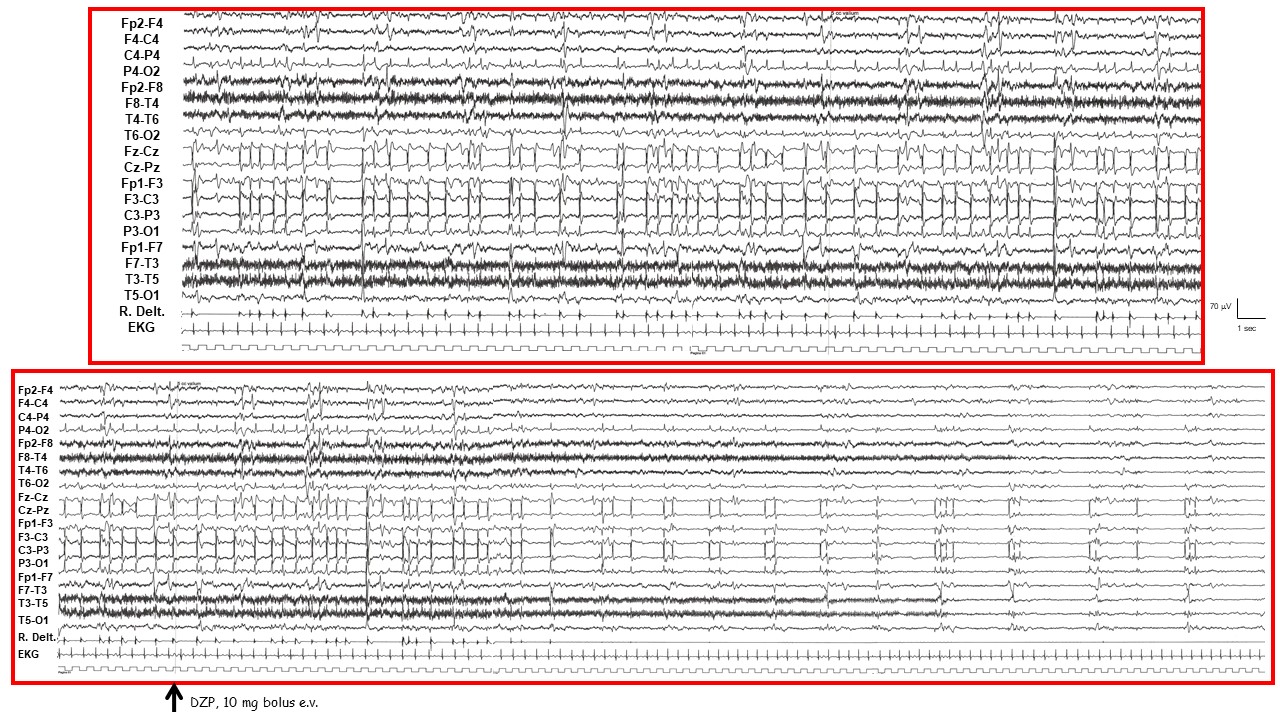

Supplement: SUPPLEMENTARY FIGURE S1 — V-EEG/PG and the pitfall of premature discontinuation (Case 1). The V-EEG/PG trace illustrates continuous paroxysmal activity prevalent over the vertex and left superior-sylvian longitudinal derivations, with a corresponding myoclonic-type muscular potential recorded over the right deltoid muscle. Following the intravenous administration of diazepam 10 mg (indicated by the arrow), this electro-clinical pattern gradually disappeared, confirming seizure termination. However, the immediate discontinuation of monitoring prevented the detection of the subsequent re-emergence of NCSE. This highlights that V-EEG/PG must be continued in high-risk patients until the patient is fully awake. [file Image_1.JPEG]

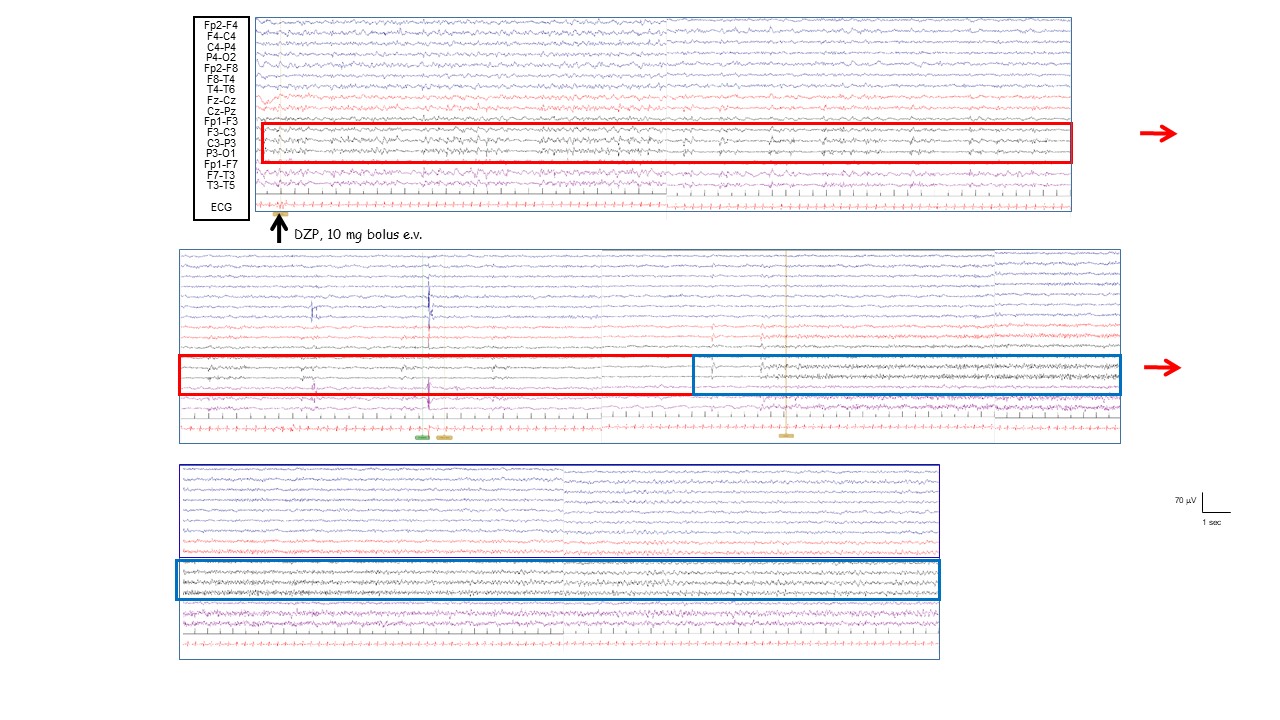

Supplement: SUPPLEMENTARY FIGURE S2 — Successful detection of NCSE via continous V-EEG/polygraphy (Case 2). The V-EEG/PG documents paroxysmal activity prevalent over the left superior-sylvian derivations (Red Box), corresponding to the right focal motor seizure. Following intravenous diazepam 10 mg, the motor seizure ceases, but the V-EEG/PG reveals persistent, continuous focal rapid and rhythmic activity in the left soprasylvian leads (Blue Box). This activity was associated with mild rightward eye deviation (seen only on synchronized video), confirming the diagnosis of Established NCSE. [file Image_2.JPEG]

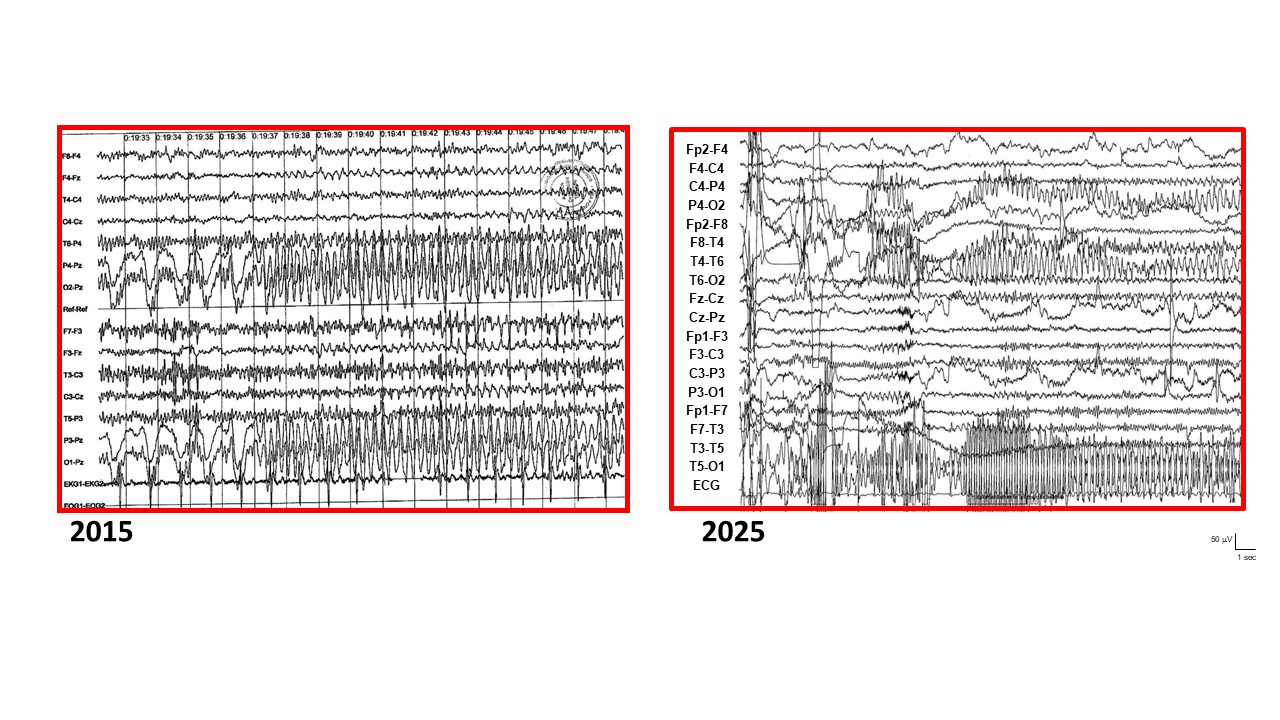

Supplement: SUPPLEMENTARY FIGURE S3 — Comparison between standard EEG (2015, left) and V-EEG/PG (2025, right) in a patient with psychogenic non-epileptic seizures (PNES). Both tracings show identical muscle artifacts during motor episodes. In 2015, the lack of simultaneous video documentation led to the erroneous interpretation of these artifacts as paroxysmal epileptiform activity, resulting in 10 years of unnecessary polypharmacy and drug-refractory “epilepsy.” In 2025, the V-EEG/PG recording definitively identified the same patterns as artifacts associated with functional movements, with a preserved background rhythm. This comparison highlights the “diagnostic trap” of movement artifacts in standard EEG. The use of V-EEG/PG was essential to correct the 10-year-old error, allow for the safe withdrawal of all anti-seizure medications, and significantly improve the patient’s quality of life. [file Image_3.JPEG]
